# Physico-Chemical Properties and Valorization of Biopolymers Derived from Food Processing Waste

**DOI:** 10.3390/molecules28196894

**Published:** 2023-09-30

**Authors:** Teresa Silvestri, Paola Di Donato, Irene Bonadies, Annarita Poli, Mariaenrica Frigione, Marco Biondi, Laura Mayol

**Affiliations:** 1 Department of Pharmacy-Pharmaceutical Sciences, University of Bari Aldo Moro, Via E. Orabona 4, 70125 Bari, Italy; teresa.silvestri@uniba.it; 2 Department of Science and Technology, University of Naples Parthenope, Centro Direzionale, Isola C4, 80143 Naples, Italy; paola.didonato@uniparthenope.it; 3 Institute of Polymers, Composites and Biomaterials, National Research Council of Italy, Via Campi Flegrei 34, Pozzuoli, 80078 Naples, Italy; irene.bonadies@cnr.it; 4 Institute of Biomolecular Chemistry, National Research Council of Italy, Via Campi Flegrei 34, Pozzuoli, 80078 Naples, Italy; annarita.poli@icb.cnr.it; 5 Department of Innovation Engineering, University of Salento, Via Arnesano, 73100 Lecce, Italy; 6 Department of Pharmacy, University of Naples Federico II, Via D. Montesano 49, 80131 Naples, Italy; mabiondi@unina.it; 7 Interdisciplinary Research Centre on Biomaterials, CRIB, University of Naples Federico II, P.l Tecchio, 80, 80125 Naples, Italy; laumayol@unina.it; 8 Department of Advanced Biomedical Sciences, School of Medicine and Surgery, University of Naples, Federico II, Via Pansini 5, 80131 Naples, Italy

**Keywords:** polysaccharides, food waste, electrospinning, nanosized fibers, renewable resources, DSC, solubility, biopolymers

## Abstract

The widespread use of synthetic plastics, as well as the waste produced at the end of their life cycle, poses serious environmental issues. In this context, bio-based plastics, i.e., natural polymers produced from renewable resources, represent a promising alternative to petroleum-based materials. One potential source of biopolymers is waste from the food industry, the use of which also provides a sustainable and eco-friendly solution to waste management. Thus, the aim of this work concerns the extraction of polysaccharide fractions from lemon, tomato and fennel waste. Characterizing the chemical–physical and thermodynamic properties of these polysaccharides is an essential step in evaluating their potential applications. Hence, the solubility of the extracted polysaccharides in different solvents, including water and organic solvents, was determined since it is an important parameter that determines their properties and applications. Also, acid-base titration was carried out, along with thermoanalytical tests through differential scanning calorimetry. Finally, the electrospinning of waste polysaccharides was investigated to explore the feasibility of obtaining polysaccharide-based membranes. Indeed, electrospun fibers are a promising structure/system via which it is possible to apply waste polysaccharides in packaging or well-being applications. Thanks to processing feasibility, it is possible to electrospin waste polysaccharides by combining them with different materials to obtain porous 3D membranes made of nanosized fibers.

## 1. Introduction

The sustainability of plastic materials is a major focus of research, industry, government, and society, and there is a need to address the negative consequences concerning end-of-life outcomes and material circularity. Challenges related to the wide diversity of materials must be tackled, and opportunities aimed at achieving a closed-loop environment need to be explored [1]. In this context, bio-based plastics, derived from renewable resources, are considered a promising alternative to petroleum-based materials due to the insufficient availability of fossil resources in the near future and the affordable low cost of renewable ones that might be consumed for biopolymer synthesis [2].

Therefore, there is a lively research interest concerning the use of natural polysaccharides as renewable sources in different fields, such as tissue engineering [3,4,5], food industry and packaging [6,7]. Besides the relevant advantages from both environmental and economic perspectives, biopolymers are, in general, biodegradable [8,9] and possess favorable toxicity profiles [10].

One potential source of biopolymers is waste from the food industry. Global population growth is leading to an increase in food demand, resulting in companies generating large amounts of untreated waste that is considered of no value [11]. This, in turn, is causing substantial pollution due to the creation of considerable amounts of waste [12,13]. In fact, most of the vegetables produced are processed for fresh consumption or to produce packaged products [14], such as juices [15], sauces [16] and liqueurs [17,18], thus generating many by-products that are used as fodder for animals or for composting [19]. A significant fraction of this waste ends up in landfills or is incinerated [20,21].

The transformation of vegetables represents an important source of profit and an excellent opportunity for the circular flow of secondary raw materials, providing an eco-friendly and sustainable solution to waste management [22,23]. The extraction of polysaccharides from food industry waste is a challenging process due to the complexity of the starting materials and the need for environmentally friendly extraction methods. Recently, green extraction methods have been developed to obtain high-quality polysaccharides with minimal environmental impact [24].

Lemons, tomatoes and fennel are commonly used in the food industry, and their waste represents an excellent source of natural polysaccharides [25]. The extraction of polysaccharides from these waste materials represents a sustainable and eco-friendly solution to waste management while also providing a renewable source of materials for various applications [26].

In this context, the aim of this work concerns the extraction of polysaccharide fractions from lemon, tomato and fennel waste. Characterizing the chemical–physical and thermodynamic properties of these polysaccharides is an essential step in evaluating their potential applications. Thus, the solubility of the extracted polysaccharides in different solvents, including water and organic solvents, was determined since it is an important parameter that determines their properties and applications. Also, acid-base titration was carried out, along with thermoanalytical tests through differential scanning calorimetry (DSC). Finally, the electrospinning of waste polysaccharides was investigated to explore the feasibility of obtaining polysaccharide-based membranes. Indeed, electrospun fibers are a promising structure/system via which it is possible to apply waste polysaccharides in packaging or well-being applications. Thanks to processing feasibility, it is possible to electrospin waste polysaccharides by combining them with different materials in order to obtain porous 3D membranes made of nanosized fibers [27,28,29].

## 2. Results and Discussion

### 2.1. Extraction of Polysaccharides from Fennel, Lemon and Tomato Waste

The use of waste biomass as a source of value-added chemicals is one main strategy for the valorization of a variety of organic residues, like those produced by the food industry. The key step in the recovery of polysaccharides from food processing waste, like the vegetable residues that we selected, is represented by chemical extraction. Chemical extraction can be implemented by means of different methods, including maceration and ultrasound- or microwave-assisted extraction. Depending on the chemical nature of the starting material and on the used techniques, the polysaccharide recovery yield and the gross chemical composition of extracted fractions can vary. As shown in Table 1, various methods are reported in the literature for the recovery of polysaccharides and fibers, and they have been applied to different waste and residual vegetable matrices [30]. The classical method, i.e., hot water temperature extraction, affords an average yield of about 6% *w*/*w*, while higher yields can be obtained by means of other methods, like those listed in Table 1.

On the basis of the results obtained in a previous study [36], here polysaccharide fractions were recovered by means of alkali maceration at room temperature prolonged for 48 h. As shown in Table 2, the polysaccharide yields from the tomato, lemon and fennel wastes ranged from 4.3 to 6.5% *w*/*w*; therefore, they were of the same order of magnitude as those obtainable with hot water. The advantages of extracting these polysaccharides via maceration with alkali rather than via the other methods listed in Table 1 are both chemical and economic since such a protocol does not rely on the use of costly reagents and requires less energy. Indeed, the increase in polysaccharide yields obtainable with other methods is coupled with some main disadvantages, for example, a high energy demand in the case of hot water extraction (with temperatures ranging from 70 to 100 °C) or supercritical fluid extraction; complex apparatuses with regard to supercritical fluids and ultrasound and microwave extraction; and the high economical costs of reagents like in the case of extraction by means of enzymes or eutectic solvents.

The extraction yield and the gross chemical composition of the raw polysaccharide fractions obtained from the lemon, fennel and tomato dried wastes are reported in Table 2. For all the polysaccharide extracts, a chemical analysis was carried out by determining the carbohydrate and protein contents in the dried extracts: as reported, all the fractions recovered after maceration showed a high carbohydrate content (Table 2) and very low amounts of protein residues.

### 2.2. Differential Scanning Calorimetry (DSC)

**Figure 1:**
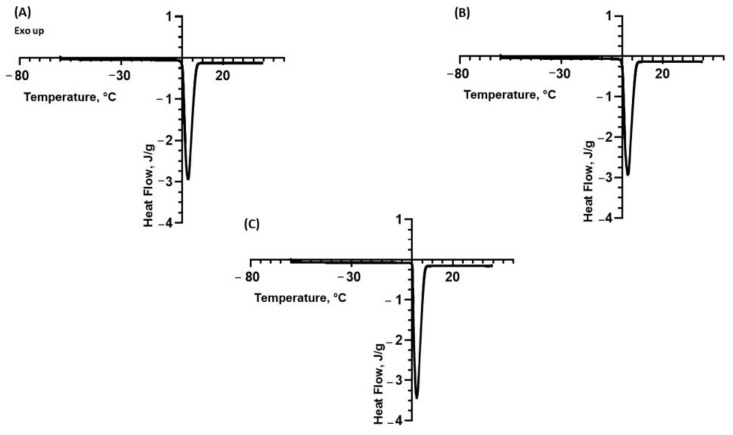
DSC thermograms of suspensions of polysaccharides extracted from (**A**) fennel at 0.1% *w*/*v*; (**B**) lemon at 0.1% *w*/*v*; (**C**) tomato at 0.1% *w*/*v*. The exothermic peaks are directed upwards.

To investigate the physico-chemical properties of the polysaccharides, a DSC analysis was performed on their aqueous suspensions at 0.1% and 1% *w*/*v*. Actually, knowledge of the thermodynamic properties of biopolymers is pivotal in defining the processing conditions and technologies to be employed. The results obtained on the suspensions of polysaccharides at 0.1% *w*/*v* extracted from the fennel, lemon and tomato are shown in Figure 1A–C, respectively.

As can be observed in Figure 1A–C, all thermograms evidenced an endothermic peak associated with an enthalpy variation deriving from water melting.

The analysis carried out on the suspension containing the polysaccharides extracted from the fennel shows an average peak temperature (Tpeak) of around 3.60 °C, an onset temperature of 0.20 °C and an enthalpy (ΔH) equal to 315 J/g. The DSC analysis of the lemon polysaccharides showed a Tpeak equal to 2.68 °C, an onset temperature equal to 0.15 °C and an ΔH of 341 J/g. Moreover, the DSC thermograms of the polysaccharides extracted from the tomato showed a Tpeak of 1.07 °C, an onset temperature of 0.12 °C and an enthalpy value (ΔH) of 349 J/g. These results are summarized in Table 3.

**Figure 2:**
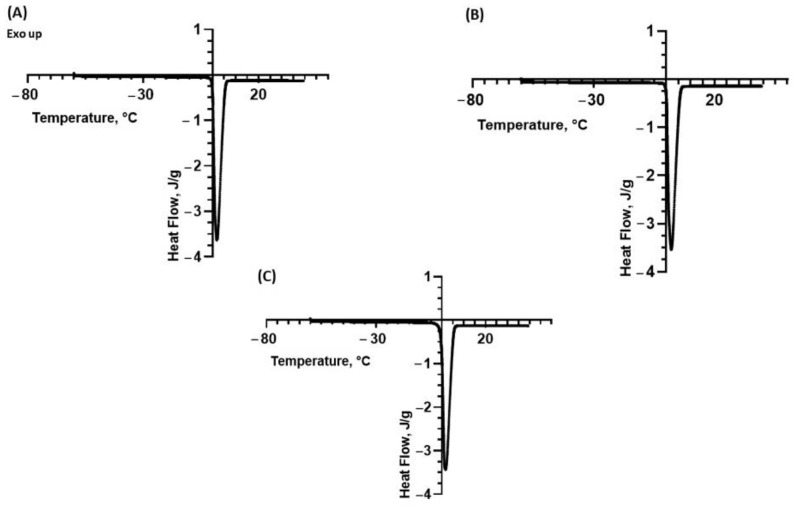
DSC thermograms of suspensions of polysaccharides extracted from (**A**) fennel at 1.0% *w*/*v*; (**B**) lemon at 1.0% *w*/*v*; (**C**) tomato at 1.0% *w*/*v*. The exothermic peaks are directed upwards.

The same DSC analyses were repeated on the polysaccharide suspensions at 1.0% *w*/*v*. The relative results are reported in Figure 2A–C, respectively.

The average peak temperature, onset temperature and ΔH values are summarized in Table 4.

**Figure 3:**
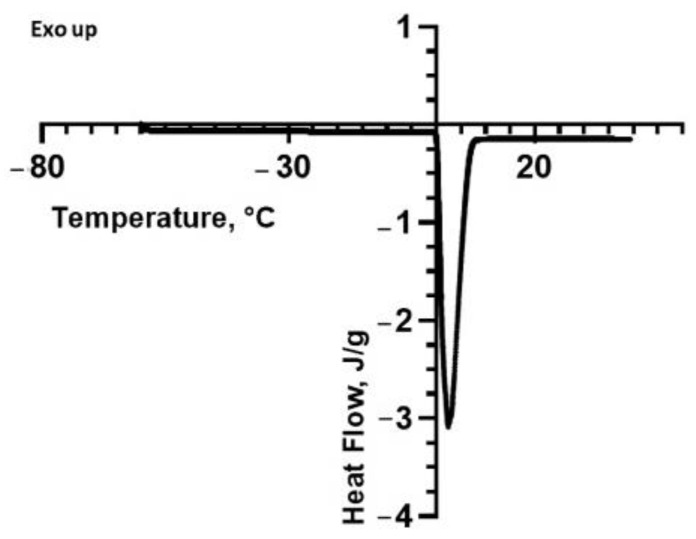
DSC thermogram of ultrapure water. The exothermic peaks are directed upwards.

By comparing the previous DSC analysis to that performed on a sample of ultrapure water, whose thermogram is reported in Figure 3, it is possible to note a variation in the endothermic peak ascribed to the presence of solubilized polysaccharides in terms of onset and peak temperature. In more detail, a careful comparison between Figure 1, Figure 2 and Figure 3 indicates that the melting peak onset is sharper in the case of ultrapure water. This suggests a slight solubilization of the polysaccharides, which is also confirmed by the decrease in the melting enthalpy in the presence of the polysaccharides. Thus, the DSC results indicate that the biopolymers extracted from the fennel and lemon are slightly more soluble than those extracted from the tomato. The variation in the melting enthalpy is more evident in the suspensions with a higher concentration.

### 2.3. Solubility Test

Polysaccharide suspensions in ultrapure water at different concentrations (0.1% *w*/*v* and 1% *w*/*v*) were prepared and kept at room temperature for two hours, under mechanical stirring. All the samples proved to be completely insoluble in water. In more detail, the suspension containing the polysaccharide extracted from the fennel had a characteristic brown color with no cake formation. The suspension containing the polysaccharide extracted from the lemon presented a yellow-ochre color appearance with a clearly visible cake. Finally, the suspension containing the polysaccharide extracted from the tomato appeared colorless and without cake.

Then, the temperature was raised to 50 °C, and, after two hours of mechanical stirring, cloudiness was observed. Conversely, without stirring, a bottom cake reformed.

Solubility tests were also performed in organic solvents (DMSO, acetone, ethanol and dichloromethane). To this end, 1 mg of each dried polysaccharide was added to 2 mL of each organic solvent. After 4 h of mechanical stirring, the formation of a sediment was observed in all the suspensions. These results are summarized in Table 5.

The tests were repeated using ultrapure water at different pH values adjusted via the addition of NaOH and HCl. In an acid environment (pH = 2), all the polysaccharides were insoluble, showing the formation of a bottom cake after 24 h of mechanical stirring. In a basic environment (pH = 12), we observed a partial solubilization of the tomato and fennel, which was indicated by the coloration of the suspension taking place. The polysaccharide from the lemon proved to be totally insoluble, leading to the formation of a bottom cake. The results are summarized in Table 6.

### 2.4. Titration

**Figure 4:**
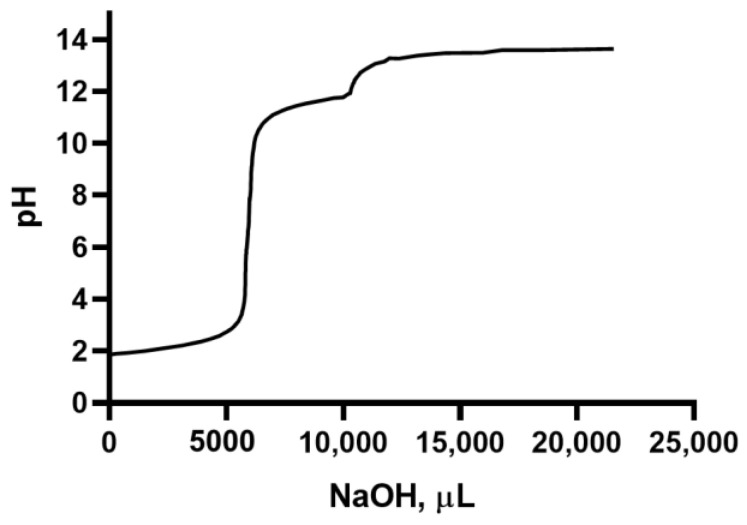
Titration curve of the suspension containing the polysaccharides extracted from fennel by adding NaOH 0.1 N as a titrating agent.

Figure 4 shows a titration curve of the suspension containing the polysaccharides extracted from the fennel obtained using NaOH as a titrating agent. As can be observed, the curve shows a rapid change in pH, which occurs at the equivalence point. This point falls in the center of the vertical section of the curve (pH ≈ 7).

**Figure 5:**
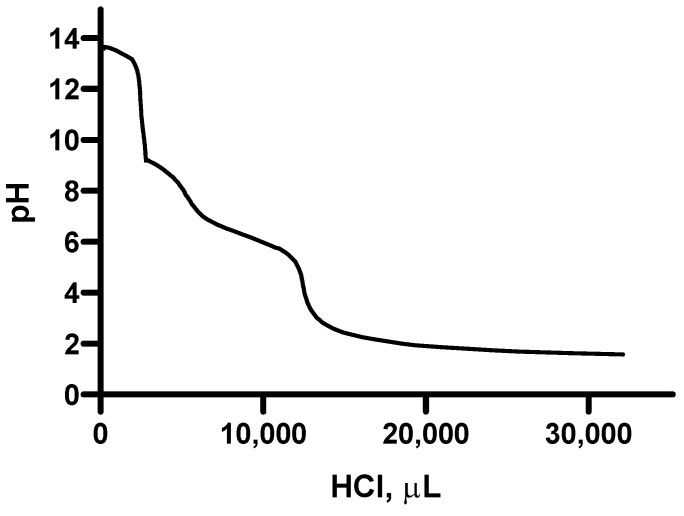
Titration curve of the suspension containing the polysaccharides extracted from fennel by adding HCl 0.1 N as a titrating agent.

By adding 0.1 N HCl as a titrating agent (Figure 5), a titration curve of a polyvalent anion of a weak polyprotic acid was obtained. Two equivalence points can be detected, corresponding to two protonation reactions occurring at pH ≈ 10 and pH ≈ 4.

It can be deduced that the analyzed polysaccharides exhibit a “buffering” behavior in an acidic environment, while the basic environment suggests that they have a weakly acidic character.

**Figure 6:**
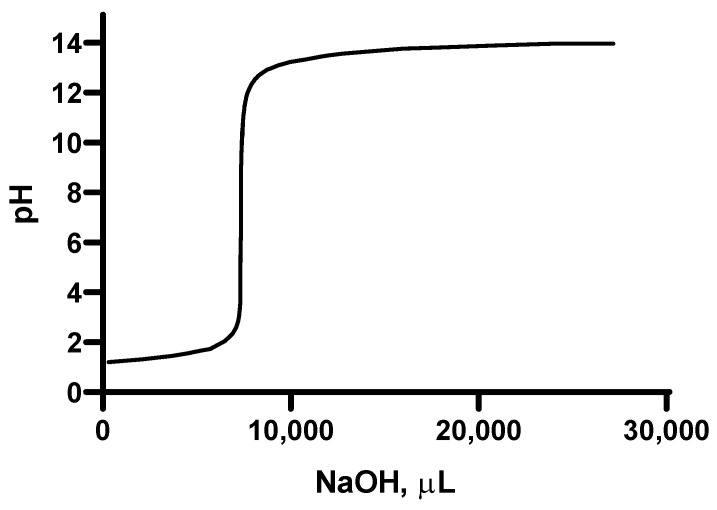
Titration curve of the suspension containing the polysaccharides extracted from lemon by adding NaOH 0.1 N as a titrating agent.

Figure 6 shows a titration curve of the polysaccharide suspensions extracted from the lemon using NaOH as a titration agent.

The curve in Figure 6 shows the equivalence point at pH ≈ 7, after the addition of about 8 mL of the titrating agent.

**Figure 7:**
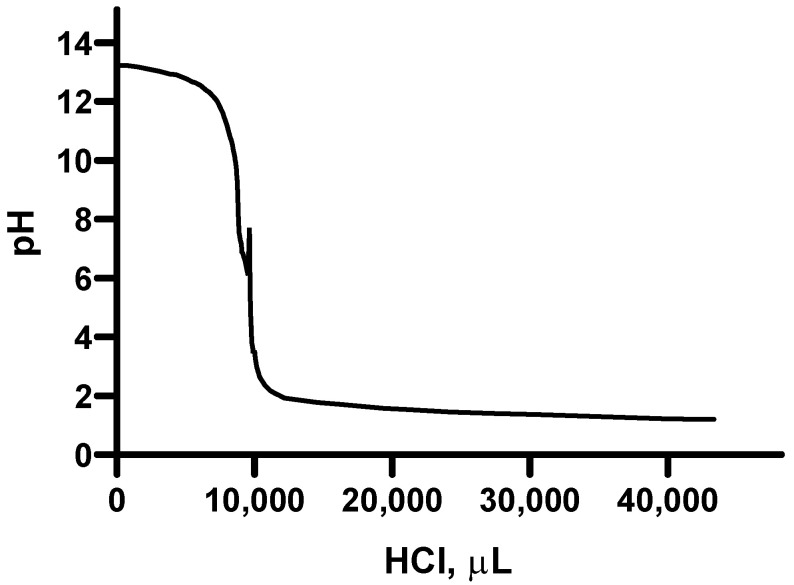
Titration curve of the suspension containing the polysaccharides extracted from lemon by adding HCl 0.1 N as a titrating agent.

In Figure 7, the titration curve of a weak base caused by the gradual addition of 0.1 N HCl is reported. Here, it can be seen that the equivalence point is reached at pH ≈ 7, after the addition of about 10 mL of the titrating agent. Thus, a moderately acid character of the analyzed polysaccharides can be deduced.

**Figure 8:**
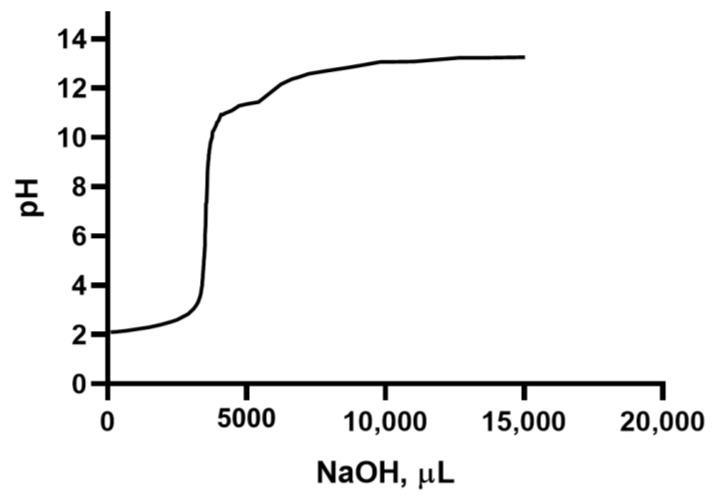
Titration curve of the suspension containing the polysaccharides extracted from tomato by adding NaOH 0.1 N as a titrating agent.

**Figure 9:**
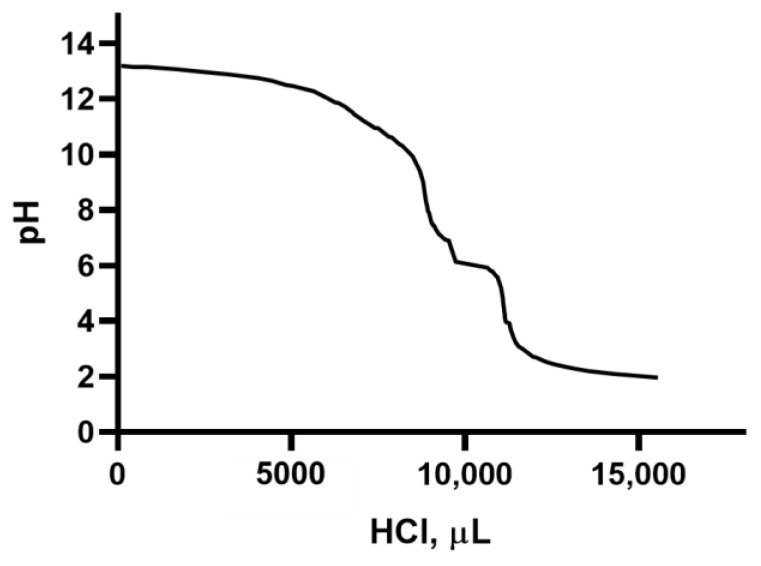
Titration curve of the suspension containing the polysaccharides extracted from tomato by adding HCl 0.1 N as a titrating agent.

In Figure 8 and Figure 9, titration curves of the suspension containing the polysaccharides extracted from the tomato are shown.

In Figure 8, it can be observed that the equivalence point was reached at pH ≈ 6.5. However, Figure 9 shows a very weak polyprotic acid behavior.

### 2.5. Fiber Formation

**Figure 10:**
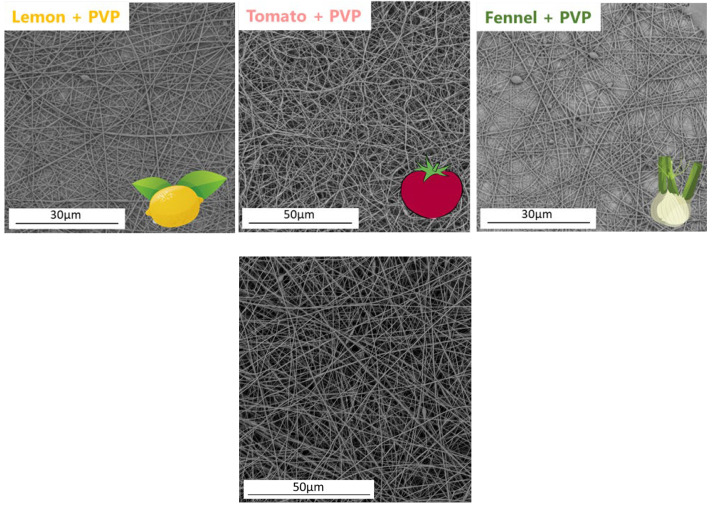
SEM micrographs of lemon + PVP, tomato + PVP, fennel + PVP and PVP electrospun nanofibers.

After optimizing the solution characteristics and electrospinning parameters, samples were collected on aluminum foil for further morphological analysis. The morphology of the fibers containing different polysaccharides was analyzed using scanning electron microscopy (Figure 10). The extract-loaded fibers did not show a different morphology from the neat PVP fibers. In fact, the addition of extracts did not change the gross appearance of the mats, as evidenced by the well spatially distributed fibers. All samples showed homogenous and uniform fibers without beads. The average diameters and corresponding standard deviations were 222 ± 39 nm (lemon), 239 ± 34 nm (tomato), 260 ± 31 nm (fennel) and 395 ± 69 nm (PVP). It was noticeable that, by adding the polysaccharides, the average diameter decreased, especially for the lemon-based fibers. This effect can be ascribed to the charged nature of the extract, whereby increasing the electrical conductivity of the loaded solution with respect to the PVP one led to a decreased diameter of the final fibers [37].

## 3. Materials and Methods

### 3.1. Materials

Tomato (*Lycopersicon esculentum* variety “Hybrid Rome”), lemon (*Citrum limon*) and fennel (*Foeniculum vulgare* var. Dulce) were kindly supplied by Fontanella Industry (Sa), Villa Massa^®^ (Piano di Sorrento, Na) and “La Gioventù” s.r.l (San Marzano sul Sarno, Salerno, Italy), respectively. A sample (200 g) of each type of waste was lyophilized (Labconco 12 Liter Cosolle Freezer Dry System) and then minced to a powder for extraction treatment. Polyvinylpyrrolidone (PVP K90, average Mw = 360,000 Da), ethanol (≥99.8%), hydrogen chloride (HCl) and sodium hydroxide (NaOH) were purchased from Sigma Aldrich (Saint Louis, MO, USA).

### 3.2. Polysaccharide Extraction and Gross Chemical Composition

Twenty grams of each powdered waste was suspended in 2 M KOH (100 mL) and macerated at room temperature under stirring for 48 h. The polysaccharide fractions were recovered as previously described [36]: briefly, the suspension was sieved (0.5 mm screen) and centrifuged at 10,000 rpm for 40 min at 4 °C. The polysaccharide fraction was recovered from the supernatant by adding (drop-wise and under stirring) cold ethanol 96% (1:1. *v*/*v*). The alcoholic mixture was stored at −20 °C overnight and then centrifuged at 10,000 rpm for 1 h at 4 °C. The resulting pellet was neutralized with HCl, dialyzed against distilled water (membrane cut-off 14,000 Da) and lyophilized. The carbohydrate content in the extracted fractions was quantified by means of Dubois’s method [38]; proteins were assayed by means of a Biorad Micro-Assay [39].

### 3.3. Differential Scanning Calorimetry (DSC)

DSC measurements were carried out using a TA DSC-Q20 instrument (TA Instrument DSC, New Castle, DE, USA) equipped with a TA Instruments DSC cooling system; indium was used for the calorimeter’s calibration. Lyophilized polysaccharide samples were suspended in ultrapure water at two different concentrations, i.e., 0.1% *w*/*v* and 1% *w*/*v*. An analysis was carried under a 50 mL min^−1^ nitrogen purge gas flow at a temperature range between −60 and 40 °C at 2 °C/min. All the measurements were performed in duplicate.

### 3.4. Solubility Tests

Solubility tests were carried out by suspending an aliquot of each lyophilized polysaccharide fraction in different solvents under the following conditions of concentration, solvent and stirring time: 0.1% *w*/*v* in ultrapure water for 2 h at both room temperature and 50 °C; 0.1% *w*/*v* in ultrapure at pH 2 or pH 12 at room temperature for 24 h; 50% *w*/*v* in dimethylsulfoxide (DMSO), acetone, ethanol or dichloromethane (DCM) for 4 h at room temperature.

### 3.5. Titration

Experiments were carried out by adding each powdered polysaccharide to ultrapure water up to a final concentration of 0.1% *w*/*v*. After the determination of the initial pH values, the solutions of either 0.1 N NaOH or 0.1 N HCl were used to derive the titration curves for all the examined waste polysaccharides.

### 3.6. Fiber Realization

Polysaccharide-based fibers were realized by using an Electrospinning Setup NF103 (MECC Co., Ltd., Fukuoka, Japan) equipped with a single nozzle and a plate collector. Starting from a solution of 10% *w*/*v* PVP in ethanol, three solutions were realized by adding the different extracted polysaccharides (PS) in a fixed amount (PVP/PS 80/20).

After the optimization of the process parameters, in order to ensure the production of defect-free fibers for further characterization, the solution flow rate was set to a fixed value of 0.2 mL/h, and the applied voltage and the nozzle-to-collector distance were set to 25 kV and 20 cm, respectively. Electrospinning was performed at room temperature and a relative humidity of 30%. Using a stationary collector covered with aluminum foil, in-plane randomly oriented fiber mats were obtained.

### 3.7. Scanning Electron Microscopy (SEM)

An FEI Phenom Desktop Scanning Electron Microscope (Thermo Fisher Scientific, Eindhoven, The Netherlands) was used to observe the morphology of the fibers. Prior to analysis, the samples went through coating with a Au−Pd alloy using a Baltec Med 020 Sputter Coater System (Balzers AG, Balzers, Liechtenstein) and were subsequently mounted on aluminum stubs. ImageJ software (National Institutes of Health, Bethesda, MD, USA) was utilized to analyze the average fiber diameter distribution.

## 4. Conclusions

To identify possible applications for the extracted polysaccharides, the physico-chemical properties of the molecules were characterized, and thermoanalytical tests were performed. The outcomes of this study revealed that the biopolymers derived from the biowastes of tomato, fennel and lemon are sparingly soluble in all the tested solvents. This led to the idea of using them as materials for the filling, plasticizing or reinforcement of other materials, such as PVP. Thus, preliminary experiments of electrospinning were carried out, and they showed that stable and smooth fibers could be obtained with the three polymers. This paves the way for continuing this experimentation to find out how biopolymer presence prompts the mechanical and biocompatibility features of the produced fibers in the view of possible application in the biomedical field.

## Figures and Tables

**Table 1:** Main extraction methods and polysaccharide yields from vegetable wastes.

Extraction Method	Average Yield (% *w*/*w*)	Ref.
Hot water	2.6–12.0	[30,31]
Ultrasound assisted	11.0–18.00	[30]
Microwave assisted	13.18–15.07	[30,32]
Enzyme assisted	15.0–19.0	[33,34]
Supercritical fluids	17.6–18.5	[30]
Eutectic solvents	8.97–11.5	[35]

**Table 2:** Extraction yield and gross chemical composition of lemon, fennel and tomato polysaccharide fractions.

Sample	Yield (mg/g Dry Waste)	Carbohydrates (% *w*/*w* Dry Extract)	Proteins (% *w*/*w* Dry Extract)
Lemon	42.9 ± 0.9	97.9 ± 0.8	2.1 ± 0.5
Fennel	65.3 ± 2.2	90.1 ± 3.7	2.3 ± 0.3
Tomato	44.1 ± 0.6	87.3 ± 4.1	1.1 ± 0.5

**Table 3:** Peak temperature, onset temperature and enthalpy of polysaccharide suspensions (0.1% *w*/*V*) and ultrapure water.

Sample	Peak Temperature, Tpeak (°C)	Onset Temperature (°C)	Enthalpy, ΔH (J/g)
Water Fennel	2.38 ± 0.15 3.60 ± 1.19	0.11 ± 0.03 0.20 ± 0.18	352 ± 5 315 ± 13
Lemon	2.68 ± 0.01	0.15 ± 0.05	341 ± 8
Tomato	1.07 ± 1.39	0.12 ± 0.01	349 ± 3

**Table 4:** Peak temperature, onset temperature and enthalpy of polysaccharide suspensions (1.0% *w*/*V*) and ultrapure water.

Sample	Peak Temperature, Tpeak (°C)	Onset Temperature (°C)	Enthalpy, ΔH (J/g)
Water Fennel	2.38 ± 0.15 2.34 ± 0.47	0.11 ± 0.03 0.63 ± 1.05	352 ± 5 342 ± 8
Lemon	2.31 ± 0.39	0.05 ± 0.07	343 ± 7
Tomato	1.82 ± 0.04	0.14 ± 0.04	349 ± 15

**Table 5:** Solubility tests in organic solvents.

Sample	Solvent	Insoluble	Poorly Soluble	Soluble
Lemon	DMSO		X	
	Acetone	X		
	Ethanol	X		
	DCM	X		
Fennel	DMSO	X		
	Acetone	X		
	Ethanol	X		
	DCM	X		
Tomato	DMSO	X		
	Acetone	X		
	Ethanol	X		
	DCM	X		

**Table 6:** Water solubility test in organic solvents at different pH values.

Sample	pH	Insoluble	Poorly Soluble	Soluble
Lemon	12		X	
	2		X	
Fennel	12			X
	2		X	
Tomato	12		X	
	2		X	

## Data Availability

The data presented in this study are available on request from the corresponding author.

## Sample Availability

Samples of the compounds are available from the authors.
